# Peroxisomes Regulate Cellular Free Fatty Acids to Modulate Mast Cell TLR2, TLR4, and IgE-Mediated Activation

**DOI:** 10.3389/fcell.2022.856243

**Published:** 2022-05-13

**Authors:** Dihia Meghnem, Edwin Leong, Marinella Pinelli, Jean S. Marshall, Francesca Di Cara

**Affiliations:** ^1^ Dalhousie Human Immunology and Inflammation Group, Department of Microbiology and Immunology, Dalhousie University, Halifax, NS, Canada; ^2^ Department of Pediatrics, Nova Scotia Health Authority IWK, Halifax, NS, Canada; ^3^ Department of Microbiology and Immunology, Dalhousie University, Halifax, NS, Canada; ^4^ Department of Pathology, Dalhousie University, Halifax, NS, Canada

**Keywords:** peroxisome, mast cell, IgE, TLR, free fatty acids

## Abstract

Mast cells are specialized, tissue resident, immune effector cells able to respond to a wide range of stimuli. MCs are involved in the regulation of a variety of physiological functions, including vasodilation, angiogenesis and pathogen elimination. In addition, MCs recruit and regulate the functions of many immune cells such as dendritic cells, macrophages, T cells, B cells and eosinophils through their selective production of multiple cytokines and chemokines. MCs generate and release multi-potent molecules, such as histamine, proteases, prostanoids, leukotrienes, heparin, and many cytokines, chemokines, and growth factors through both degranulation dependent and independent pathways. Recent studies suggested that metabolic shifts dictate the activation and granule content secretion by MCs, however the metabolic signaling promoting these events is at its infancy. Lipid metabolism is recognized as a pivotal immunometabolic regulator during immune cell activation. Peroxisomes are organelles found across all eukaryotes, with a pivotal role in lipid metabolism and the detoxification of reactive oxygen species. Peroxisomes are one of the emerging axes in immunometabolism. Here we identified the peroxisome as an essential player in MCs activation. We determined that lack of functional peroxisomes in murine MCs causes a significant reduction of interleukin-6, Tumor necrosis factor and InterleukinL-13 following immunoglobulin IgE-mediated and Toll like receptor 2 and 4 activation compared to the Wild type (WT) BMMCs. We linked these defects in cytokine release to defects in free fatty acids homeostasis. In conclusion, our study identified the importance of peroxisomal fatty acids homeostasis in regulating mast cell-mediated immune functions.

## Introduction

Mast cells (MCs) are highly specialized cells able to respond to a large panel of stimuli (Theoharides et al., 2019). MCs are characterized by highly metachromatic granules with potential for different routes of release (Wernersson and Pejler, 2014; Jain et al., 2019). MCs can respond rapidly to stimuli by releasing granules containing antimicrobial cytotoxic mediators such as serine protease, histamines, proteoglycans and lysosomal enzymes or performing *de novo* synthesis independent of degranulation of reactive oxygen species (RO) and cytokines (Moon et al., 2014). MCs are also endowed with complex lipid droplets (Dichlberger et al., 2013) making them an important source of various lipid mediators (eicosanoids) such as leukotrienes and prostaglandins which are important players in immune cell activation and recruitment (Boyce, 2005). In addition to lipid production, MCs have been shown to respond to lipid mediator stimulation (Abdel-Majid and Marshall, 2004; Wang and Kulka, 2015; Hagemann et al., 2019).

Changes in metabolism have recently been identified as a mechanism that supports MCs activation such as IgE mediated degranulation (Mendoza et al., 2021). Several studies have shown the importance of lipid metabolism in MCs that goes beyond the production of lipid mediators. In fact, high-fat diet or chronic insulin exposure led to a lipid accumulation and altered degranulation in MCs (Greineisen et al., 2015; Aldan et al., 2019). However, how lipid metabolism supports MCs activation and regulates their distinct activities such as degranulation and/or cytokine release is largely unexplored and represents an important area of investigation to unravel how these essential innate immune cells are regulated.

Peroxisomes are specialised organelles for metabolism found across all eukaryotes. Peroxisomes have a pivotal role in lipid metabolism and in detoxification of ROS and reactive nitrogen species. They also contribute to the metabolism of polyamines, carbohydrates and amino acids (Wanders and Waterham, 2006; Fransen et al., 2012; Smith and Aitchison, 2013; Liu et al., 2019)**.** There is substantial evidence that peroxisomes actively contribute to cell signaling and that their function is required for human health (Beach et al., 2012; Braverman et al., 2013; Braverman et al., 2013; Fransen et al., 2013; Trompier et al., 2014; Colasante et al., 2015). Recent evidence corroborated a role for peroxisomes in modulating immune responses (Dixit et al., 2010; Di Cara et al., 2017; Di Cara, 2020). Indeed, peroxisomes were first described to have an important role during viral infections serving as signal platforms for mitochondrial antiviral signaling (MAVS) proteins and induction of interferon responses (Sychev et al., 2017; Xu et al., 2017; Cook et al., 2019; Merkling et al., 2019). In recent years, substantial evidence has shown the importance of peroxisome metabolism in macrophage activation and phagocytosis (Boncompain et al., 2014; Di Cara et al., 2017; Eguchi et al., 1979; Vijayan et al., 2017). Further studies showed the importance of peroxisome-derived ether lipids in natural killer T (NKT) cell thymic development (Brutkiewicz and Dent, 2012; Facciotti et al., 2012). Thus, peroxisomes contribute to drive signaling pathways in innate and adaptive immune responses through metabolites such as ROS and lipids such as fatty acids. The metabolism of fatty acids (FAs) is a major source of biological lipids that form cell membranes and regulate inflammatory processes (Puertollano et al., 2001; Sadik and Luster, 2012; Dowds et al., 2014; Hubler and Kennedy, 2016). FAs are precursors to phospholipids (PLs), sphingolipids (SLs), triglycerides (TAGs) and eicosanoids, which have critical roles in the activation and function of macrophages, invariant NKT cells (Lim et al., 2003; Miao et al., 2014; Bettencourt and Powell, 2017). Likewise, the PL precursor, phosphatidic acid (PA), regulates the mammalian target of rapamycin (TORC1)-dependent production of pro-inflammatory cytokines in macrophages (Lim et al., 2003).

MCs are essential innate immune cells. Beyond their activities in allergic disease, MCs play a crucial role in host defense (Marshall, 2004; Abraham and St John, 2010) and cancer immunity (Oldford et al., 2010; Komi and Redegeld, 2020; Hanes et al., 2021). Mast cell degranulation mechanisms are well studied but much less is known about how lipid metabolism regulates MCs functions. Here we determined the requirement for peroxisomes in regulating distinct immune functions in MCs. We probed the need for functional peroxisomes in mounting Toll like receptor(TLR)2 and 4, IgE-mediated activation of Bone marrow-derived mast cells (BMMCs) extracted from wildtype (WT) mice and mice carrying a global mutation for *Peroxin2*, a gene that encodes for an ubiquitin ligase essential for the biogenesis of peroxisomes in cells and therefore its mutation leads to cells with not functional peroxisomes (Faust and Hatten, 1997; Smith and Aitchison, 2013). Our work demonstrated a role for peroxisomes in modulating cellular free fatty acids (FFAs) to regulate TLR and IgE-dependent secretion of cytokines in MCs. In stimulated WT MCs, peroxisome number increases, contributes to cellular FFAs homeostasis and support cytokines release. Of note, peroxisomes appeared dispensable for IgE-mediated degranulation. Taken together our report provides evidence of a requirement for peroxisome to control cellular lipid metabolism for distinct MC immune functions. Defining the role of peroxisomal metabolism in MCs may uncover new avenues of treatment for immune disorders and requires greater insight into the function of specific metabolic pathways involved in immune responses.

## Methods

### *Pex2* Mutant Mice

The *Pex2* Mutant Mouse Strain used was 129S6.129-*Pex2* ^*tm1Plf*^/Mmmh(Null allele) (Faust and Hatten, 1997) and was obtained from the Mutant Mouse Resource and Research Centre (MMRRC) supported by the NIH. The mice used for this experiment were *Pex2* ^*+/+*^, *Pex2* ^*−/−*^, *Pex2* ^*+/−*^. Homozygous null mutant strains showed no *Pex2* transcript and protein. Homozygous mutants in this congenic strain show variable embryonic lethality, starting at ∼E11. Approximately 20% of homozygotes survive to birth but are hypotonic, do not feed and die on the day of birth. Homozygous mutants that survive in the postnatal period are obtained by mating congenic 129S6.129-*Pex2* ^*tm1Plf*^ +/- mice with wild-type Swiss Webster strain mice. F1-*Pxmp3* ^*tm1Plf*^+/- hybrids (designated Sw129) are then intercrossed to obtain Sw129-*Pxmp3* ^*tm1Plf*^−/− (indicated in the text as *Pex2* ^*−/−*^) mice.

Colonies were maintained as stable inbred lines in the Swiss Webster and 129SVEV background under approved animal protocol 21-023, abiding by the standards of the Canadian Council on Animal Care.

### Mast Cell Culture

BMMCs were generated from SWR/J and *Pex2* ^*−/−*^ mice according to the method of (Tertian et al., 1981). After at least 4 weeks of culture, the purity of mast cells was evaluated based on the expression of the high-affinity IgE receptor, also known as FcεRI and tyrosine-protein kinase cKIT (Cluster of differentiation, CD117). Cells were used at >98% of purity and consistently contained metachromatic granules.

### Polymerase Chain Reaction

Total RNA was extracted using the RNeasy Plus Mini Kit (Qiagen, Mississauga, Canada). Genomic DNA was depleted, and complementary DNA was amplified using the Platinum Taq Reverse Transcription Kit (Wisent). *Pex2* gene was amplified using HiFi Platinum Taq DNA kit (Thermofisher) and the following primers forward (5′-TGA​AGG​AAC​CAC​TTA​GAA​ATT​ACA​GA) and reverse (5′-CCA​GGG​CCT​TAT​TCA​GTT​CA). Samples were loaded onto a 2.5% agarose gel (with ethidium bromide) in TAE and imaged using chemiDoc imaging system (Biorad).

### Toluidine Blue Staining

Cytospins of mast cells were briefly fixed in Carnoy’s fixative then rinsed in water and 0.033N HCl. Cells were then stained with Toluidine blue (pH 0.3) overnight then rinsed before drying and mounting in DPX (Sigma) for imaging with Mantra 2TM at ×40 magnification.

### Degranulation Assessment

BMMCs (2 × 10^6^/ml) in modified HEPES-Tyrode’s buffer were treated for 15 min with increasing doses of TNP-BSA (Trinitrophenyated-Bovine serum albumin) (Bioresearch Technologies) or calcium ionophore A23187 (Sigma) as a positive control. The level of degranulation was assessed *via* β-hexosaminidase release according to the method of Schwartz et al. (Schwartz et al., 1979). The percentage of β-hexosaminidase release was calculated as follow:

% of release ={(O.D supernatant−O.D control)÷[(O.D supernatant−O.D control)+(O.D pellet−O.D control)]}X100

### Mast Cell TLR and IgE Activation

Prior to all activations, BMMC were ‘‘rested’’ overnight in modified mast cell growth medium, with 3 ng/ml mIL-3 (Peprotech) or without PGE_2_ (Tocris). For analysis of cytokine production, cells were washed twice and resuspended in medium consisting of RPMI 1640 with 1% FBS, 15 mM HEPES and 3 ng/ml rmIL-3 and 100 μg/ml of soybean trypsin inhibitor. For all activations, cells at 1 × 10^6^/ml were incubated with Pam3-CSK4-KKKK (L2000, EMC microcollection) at 50 μg/ml or LPS (Sigma) at 50 μg/ml and A23187 (Sigma) at 0.5 µM for 24 h at 37°C. For IgE activation, BMMCs were sensitized with anti-TNP (Trinitrophenol phosphate) IgE overnight. Cells were then rinsed and treated with 10 ng/ml of TNP-BSA for 30 min, then supernatants were removed, and cells were cultured for a further 24 h. For mechanistic studies, cells were treated with 100 μg/ml of niacin (Sigma Aldrich) for 48 h or with 2.5 uM thioridazine for 1 h prior to IgE activation. Supernatants were removed, and cells were cultured for a further 24 h. Cell-free supernatants were collected and assayed for IL-6 (Peprotech), IL-13 (Peprotech) and TNF (Invitrogen) by ELISA from sources indicated.

### Free Fatty Acid Assessment

One million BMMCs per genotype and under each condition was sensitized with anti-TNP as described above and treated with 10 ng/ml of TNP-BSA for 24 h. Supernatants were removed, and cells were analyzed for fatty acid accumulation using free fatty acid quantification kit (Sigma) according to manufacturer’s recommendations.

ELISA: Levels of IL-6 (Peprotech), IL-13 (Peprotech) and TNF (Invitrogen) in supernatants were assessed according to the manufacturer recommendations.

### Fluorescence Microscopy

Cells were fixed in 4% paraformaldehyde in PBS for 30 min and then incubated for 1 h at room temperature in 5% normal goat serum (Sigma) and for 16 h at 4°C with primary antibody at 1:100 dilution in 5% normal goat serum. Appropriate Alexa Fluor secondary antibodies (anti-rabbit secondary antibodies, were from Abcam) were then used at 1:1000 dilution in 5% normal goat serum. After 4 washes in PBST (PBS +0.1% (v/v) Triton X- 100), cells were mounted in DAPI Pro-Gold Antifade Reagent (Thermo Fisher) and imaged using a ×100 oil immersion objective (NA = 1.4) mounted on an Zeiss800 confocal microscope (Zeiss) or using a Zeiss AxioObserver LSM 880, 100 × 1.4 oil plan-Apochromat lens. Primary antibody was rabbit anti-SKL antibody was previously described (Szilard et al., 1995).

Flow cytometry: Maturation of *Pex2* ^*−/−*^ and WT BMMC were assessed by flow cytometry. Antibodies to CD117 (Clone 2B8, Biolegend), FceR1(Clone MAR-1, Invitrogen) were used to assess the maturation of BMMC cells. Fc receptors were first blocked with anti CD16/CD32 (Clone 93, eBisocience) for 10 min. Combinations of the two anti-CD117 and anti-FceR1 fluorescently tagged antibodies (at manufacturers recommended dilutions) were added to each sample for 30 min at 4°C. For assessment of IgE binding, BMMCs were sensitized with anti-TNP (Trinitrophenol phosphate) IgE overnight. Cells were then rinsed then stained with fluorescently tagged anti-IgE antibody (ClonePME-1, Biolegend) or with isotype control rat IgG2b (Clone G0114F7, Biolegend) for 30 min at 4°C.

After stainings, cells were washed twice with PBS supplemented with 2% Fetal Calf Serum (FCS) (Gibco) then fixed with PBS containing 1% paraformaldehyde for 30 min 4°C before analysis on BD FACSCelesta™ (BD). Data were analyzed using FlowJo Version 10 software (BD).

### Viability Assay

BMMCs were resuspended at a density of 1 million cells per mL in activating media and seeded into a 24-well plate. BMMCs were stimulated in duplicates with either activation media, LPS (50 μg/ml), Pam3CSK4 (50 μg/ml), or thiorizi dine (2.5 uM) for 24 h. BMMCs were then washed in PBS prior to staining with fixable viability dye Efluor 450 for 20 min at 4°C then rinsed before fixation in 1% paraformaldehyde, and acquired on the FACS Canto II flow cytometer. Data were analyzed using FlowJo Version 10 software (BD).

### Quantification and Statistical Analysis

Statistical analyses were performed using a non-parametric *t*-test comparing between *Pex2* ^*−/−*^ and WT BMMCs. * *p* < 0.05, ** *p* < 0.001, *** *p* < 0.0001 ns: not significant. All results are represented as the mean of at least 3 independent experiment ± Standard error of the mean.

### Quantification of SKL-Puncta

Average number of puncta per cell were calculated using ImageJ software, applying the following steps to each image:

1—We opened stacks image.

File -> Open…

2—We filtered to remove noise.

Process -> Filters -> Gaussian Blur…

3—We subtracted background.

Process -> Subtract Background…

(the box marked “Light Background” was unticked).

4—We clicked on “Image”

Color -> Split the channels …

In this step, brightness and contrast were adjusted, and setting were applied to all stacks.

5—We performed threshold image.

Image -> Adjust -> Threshold…

(We selected: Apply it to all stacks).

Box labeled “Dark Background” was ticked. We adjusted the sliders so that features were red colored, but the rest of the image was not. Then we clicked “Apply” button. This replaced grayscale image with an “8-bit binary image.” All “red” pixels were converted to a value of “255,” while all non-red pixels were given a value of “0.”

6—We filled in any holes in the nuclei.

Process -> Binary -> Fill Holes

7—We separated “Touching” puncta.

Process -> Binary -> Watershed.

Process -> Find Edges.

8—We performed the analysis.

Analyze -> Analyze Particles…

In this dialog box the algorithm started to include or exclude puncta based on their attributes. “Size” smaller than 0.1 mm and larger than 1 mm “Circularity” set range: 200-1.

## Results

### Lack of Functional Peroxisomes Does Not Alter Mast Cell Morphology or Degranulation But Reduces Cytokine Release

To study the requirement of peroxisomes for MCs function, we assayed BMMCs from wildtype and *Peroxin2* null mutant mice (*Pex2* ^*−/−*^) (Supplementray Figure S1A) (Faust and Hatten, 1997). We confirmed the presence of peroxisomes by performing indirect immunofluorescence (IF) using an antibody against the C-terminal Peroxisome Targeting Sequence Type 1 Ser-Lys-Leu (SKL), the canonical marker for peroxisomal matrix proteins (Szilard et al., 1995).

**FIGURE 1:**
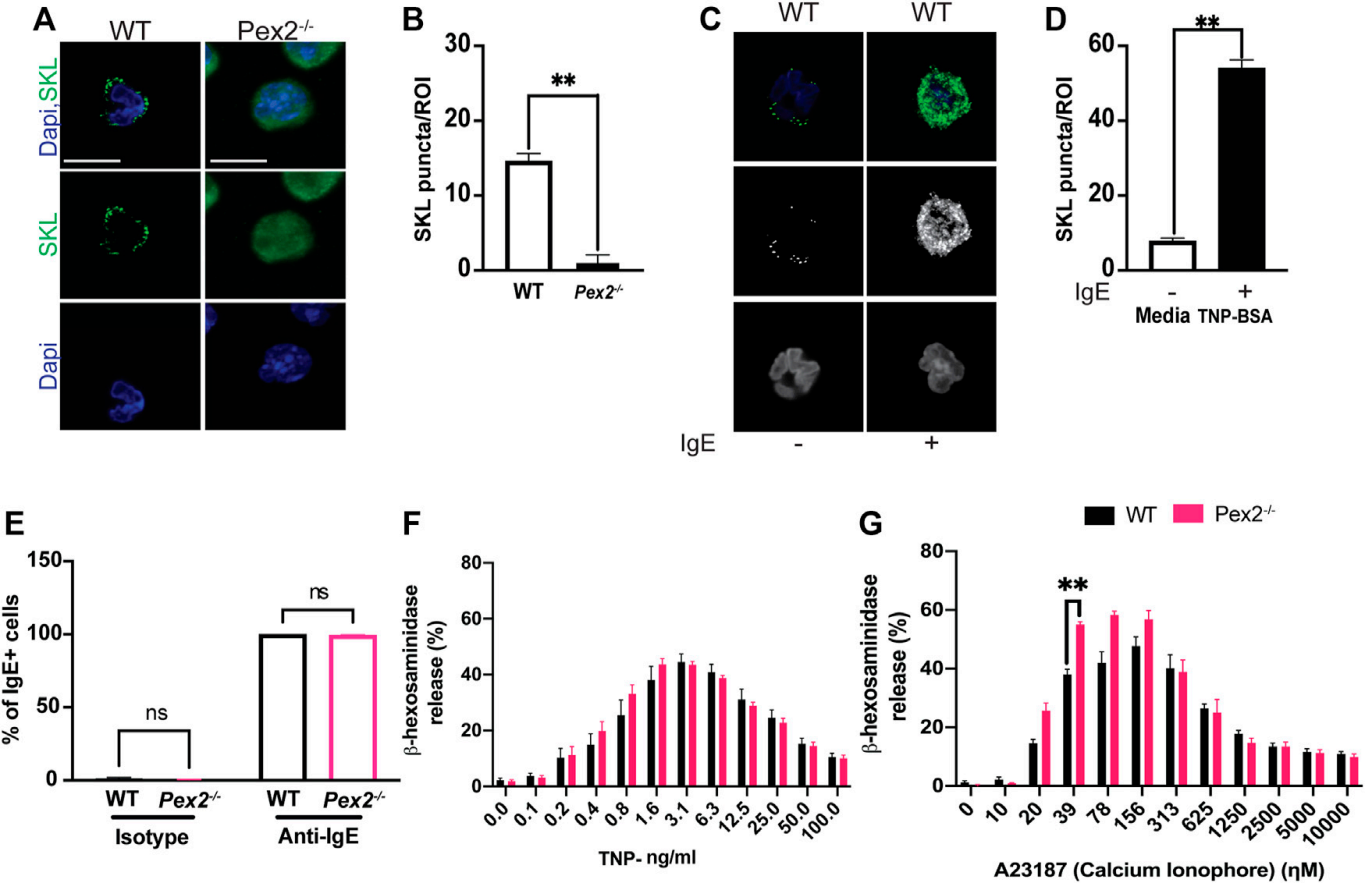
Peroxisome do not alter MCs granularity and degranulation: **(A)** WT and *Pex2* ^*−/−*^ BMMCs were stained for peroxisome (SKL, Green) and nuclei (Dapi, Blue). Scale bar, 10 µm. **(B)** Peroxisomes number was defined by automated counting of SKL-positive puncta per region of interest (ROI). The graph bars represent the number of SKL-positive puncta in stack z = 3. N = 25 cells. For each cell 22 stacks were acquired. **(C)** Indirect immunofluorescence of WT and *Pex2* ^*−/−*^ BMMCs were stained for peroxisome (SKL, Green) and nuclei (Dapi, Blue). Scale bar, 10 µm. **(D)** Peroxisomes number was defined by automated counting of SKL-positive puncta. The graph bars represent the number of SKL-positive puncta in stack z = 3. The graph bars represent the number of SKL-positive puncta in stack z = 3. N = 25 cells. For each cell 22 stacks were acquired. **(E)** WT and *Pex2* ^*−/−*^ BMMCs were tested for their ability to bind to the anti-TNP specific IgE by flow cytometry. **(F)** Percentage of beta -h exosaminidase degranulation was assessed upon 15 min of TNP-BSA IgE-mediated degranulation and **(G)** A23187 calcium ionophore-mediated degranulation. Graphs represent the average of three independent experiments ±SEM. Statistical analyses were performed using a non-parametric *t*-test comparing between *Pex2* ^*−/−*^ and WT BMMCs. ** *p* < 0.01, ns: not significant.

WT MCs showed SKL-positive puncta while intense diffuse staining was observed in *Pex2* ^*−/−*^ MCs confirming that the MCs from *Pex2* mutant mouse do not form functional peroxisomes due to defects in peroxisomal protein import into the matrix (Smith and Aitchison, 2013) (Figures 1A,B, Supplementary Figure S1A; Supplementary Data Sheet S1, S2). Assessment of maturation based on the expression of FceR1 and CD117 by flow cytometry showed no differences between the two cell types (Supplementary Figure S1B). *Pex2* ^*−/−*^ BMMCs exhibited a similar morphology and granularity compared to the WT BMMCs (Supplementary Figure S1C). All together these observations indicated that absence of peroxisomes did not alter maturation, morphology, or granule content of MCs.

Through IgE-mediated degranulation, MCs hold a key role in allergic disease and host defence against several parasites. Peroxisomes are known to proliferate and increase during responses to viral infection (Cook et al., 2019; Knoblach et al., 2021). To assess the role of IgE-mediated activation and peroxisomes we measured peroxisome numbers in WT BMMCs when stimulated with IgE. Indirect IF followed by automated quantification showed an increase in SKL-positive puncta in stimulated MCs (Figures 1C,D, Supplementary Data Sheet S3, S4) indicating that an increase in cellular peroxisomes occurs during the IgE mediated MC response. We next assessed whether an absence of peroxisomes altered IgE/antigen-induced degranulation, assessed via a β-hexosaminidase release. Peroxisome biogenesis defects caused by mutations in *Pex2* have been linked to lipid metabolic defects (Faust and Hatten, 1997; Faust, 2003) thus, affecting the lipid milieu of the cell membrane (Schrader et al., 2020). In fact, an altered membrane lipids environment was reported to compromise signaling (Lodhi et al., 2015) in multiple cell types including immune cells (Lodhi et al., 2015; Di Cara et al., 2019). Thus, we first assessed the ability of WT and *Pex2* ^*−/−*^ BMMCs to bind IgE by flow cytometry. WT and *Pex2* ^*−/−*^ BMMCs demonstrated equivalent IgE binding (Figure 1E and Supplementary Figure S1D). Next, BMMCs were loaded with Anti-TNP IgE overnight then crosslinked with a dose range of TNP-BSA antigen or treated with calcium ionophore A23187 allowing degranulation for 15 min. *Pex2* ^*−/−*^ BMMCs displayed similar degranulation potential compared to their WT counterparts (Figure 1F). When treated with calcium ionophore A23187, *Pex2* ^*−/−*^ BMMCs demonstrated a higher percent degranulation at lower A23187 doses (Figure 1G). Overall, lack of functional peroxisomes did not adversely affect mast cell degranulation.

### Peroxisome Supports Cytokine Release Upon TLRs and IgE Activation in Mast Cells

**FIGURE 2:**
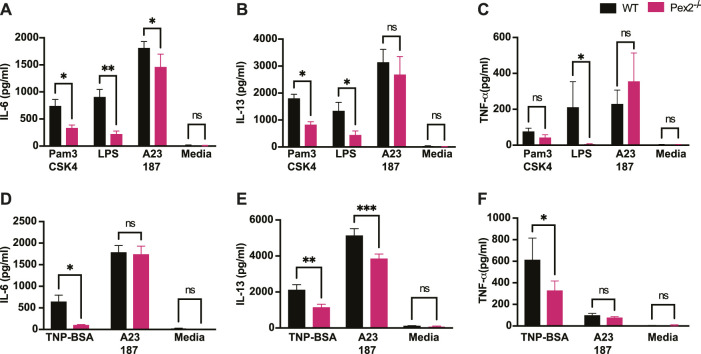
Absence of peroxisome leads to decreased MCs cytokine release upon TLRs and IgE-mediated activation: WT and Pex2−/− BMMCs were treated with TLRs agonist or TNP-BSA for 24 h and cytokines amounts were measured by ELISA. **(A)** IL-6, **(B)** IL-13 and **(C)** TNF-α production after TLR2 agonist Pam3CSK4, TLR4 agonist LPS and calcium ionophore A23187 treatments. **(D–F)** Same readouts were measured after IgE crosslinking with TNP-BSA treatment for 24 h. The media columns in each graph represent the baseline level detected for each cytokine. The graphs represent the average of three independent experiments ±SEM. Statistical analyses were performed using a non-parametric t-test comparing between Pex2^−/−^ and WT BMMCs. *p < 0.05, **p < 0.001, ns: not significant.

It is well established that MCs have degranulation-independent pathways which allow the production of cytokines and chemokines independent of classical degranulation (Leal-Berumen et al., 1994; Moon et al., 2014). Beyond their role in allergy, MCs are key player in responses to pathogens (Marshall, 2004; Abraham and St John, 2010). MCs express and respond via Toll like receptors (TLRs) to several bacterial or viral products (McCurdy et al., 2003; Agier et al., 2018). We thus asked whether peroxisome function is necessary for cytokine release upon TLR2 or TLR4 stimulation induced by Pam3CSK4 (Pam3) and *E.coli* lipopolysaccharide (LPS) respectively. We assessed whether the lack of functional peroxisomes affected TLR and IgE-mediated interleukin 6 (IL-6), interleukin 13 (IL-13) and Tumor Necrosis Factor (TNF) production after 24 h stimulation. Our data showed that IL-6 (Figure 2A) and IL-13 (Figure 2B) were produced in response to TLR2 and TLR4 stimulation in WT BMMCs but production of both cytokines was significantly reduced in *Pex2* ^*−/−*^ cells. On the other hand, TNF production was significantly reduced only upon LPS treatment in *Pex2* ^*−/−*^ compared to WT MCs (Figure 2C). Interestingly, while IgE-mediated degranulation was not affected by the absence of functional peroxisomes, cytokine production was markedly reduced in *Pex2* ^*−/−*^ BMMCs upon IgE stimulation followed by TNP-BSA treatment (Figures 2D–F). Additionally, IL-13 secretion was lower in *Pex2* ^*−/−*^ BMMCs after IgE stimulation followed by treatment with both TNP-BSA or with the calcium ionophore A231287 (Figure 2E). We tested the secretion of other cytokines such as interleukin 5 (IL-5), Granulocyte-macrophage colony-stimulating factor (GM-CSF) and Chemokine (C-C motif) ligand 3 (CCL3) (Supplementary Figure S2A–C). A23187 selectively induced CCL3 production which was significantly decreased in *Pex2* ^*−/−*^ compared to the WT BMMCs (Supplementary Figure S2C). All together these results indicated a requirement for peroxisomes in MCs activation in response to IgE-mediated, TLR2 or TLR4 stimulation.

### Peroxisome Regulates Free Fatty Acid Metabolism During MCs Activation

**FIGURE 3:**
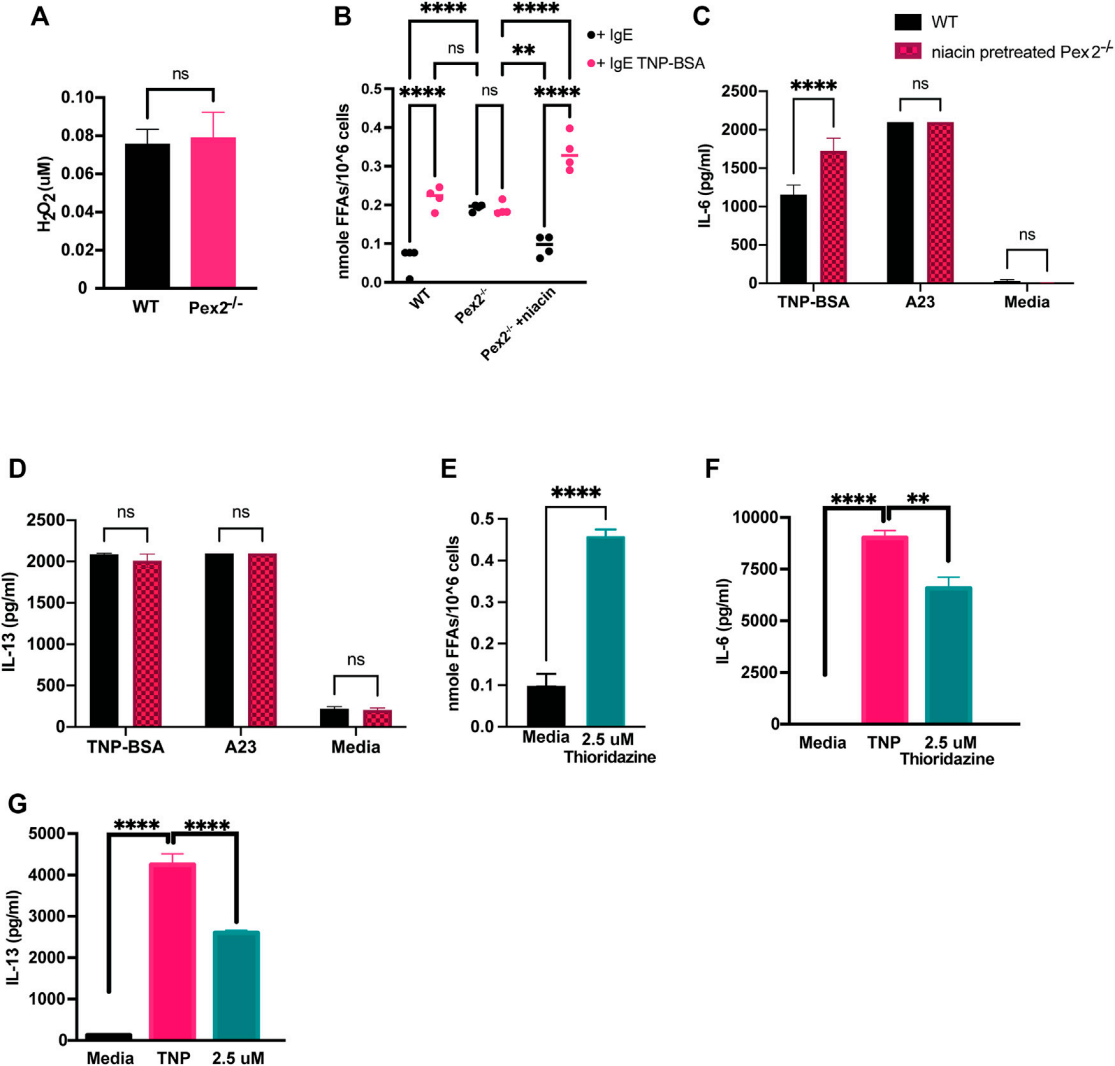
Defects in peroxisomal fatty acid metabolism affects cytokines release in MCs: **(A)** WT and Pex2^−/−^ BMMCs pellets were assessed for the level of H2O2 and **(B)** free fatty acids at baseline and after 24 h IgE mediated activation with TNP-BSA in WT, Pex2^−/−^ and niacin-treated Pex2−/− BMMCs. **(C,D)** Concentrations of IL-6 and IL-13 were assessed after 48 h of niacin treatment followed by 24 h IgE or A23 activation. **(E)** Free fatty acids amounts measured in WT BMMC at baseline and after 24 h of treatment with thioridazine. **(F–G)** Effect of free fatty acid metabolism inhibition on IgE-mediated IL-6 and IL-13 release was assessed in C57BL/6 BMMCs. The media column in graphs C-G represent the baseline level detected for each cytokine. The graphs represent the average of three independent experiments ±SEM. Statistical analysis was performed using a non-parametric *t*-test comparing between Pex2^−/−^ and WT BMMCs. **p < 0.001, ***p < 0.0001, ****p < 0.00001, ns: not significant.

Peroxisomes are highly conserved organelles and play a pivotal role in lipid metabolism and ROS such as hydrogen peroxide (H_2_O_2_) catabolism (Liu et al., 2019). Both ROS and lipids are important mediators in cellular signaling in immune cells (Di Cara, 2020; Di Cara et al., 2017; 2019). H_2_O_2_ is a permeable and diffusible molecule involved in inter- and intracellular signaling during host defense (Bedard and Krause, 2007; Blander and Sander, 2012). We measured the cellular amount of H_2_O_2_ in *Pex2* ^*−/−*^ and WT BMMCs. At rest, BMMCs lacking peroxisome function exhibited similar amounts of H_2_O_2_ (Figure 3A) as WT BMMCs.

Different studies have shown that the metabolism of fatty acids (FAs) is a major source of biological lipids that form cell membranes and regulate inflammatory functions (Dowds et al., 2014; Hubler and Kennedy, 2016; Nath et al., 2022; O'Neill et al., 2016; Puertollano et al., 2001; Sadik and Luster, 2012). Peroxisomes contribute to the homeostasis of FAs in the cell (Wanders and Waterham, 2006; Lodhi and Semenkovich, 2014) and we probed whether free fatty acids (FFAs) are altered in MCs in absence of peroxisomes, affecting cytokine release. We measured cellular FFAs and observed a significant accumulation of FFAs in *Pex2* ^*−/−*^ BMMCs compared to WT BMMCs, at rest (Figure 3B). Interestingly, we observed that IgE stimulation triggers a significant increase of FFAs in WT BMMCs while the level remained unchanged in IgE stimulated *Pex2* ^*−/−*^ (Figure 3B). These results indicated that IgE-mediated stimulation triggers an increase in cellular FFAs in MCs while lack of functional peroxisomes affects FFA metabolism and turnover at rest and during IgE-mediated activation.

To explore the link between FFAs and MCs activation, we used Niacin a vitamin B3 shown to reduce FFAs in plasma, macrophages and adipocytes by inducing anti-lipolytic effects (Tunaru et al., 2003; Nath et al., 2022). Human MCs have been shown to respond to niacin treatment by prostaglandin D2 (PGD_2_) production (Papaliodis et al., 2008). We hypothesised that the accumulation of FFAs in WT BMMCs was hindering cytokine release under IgE-mediated stimulation conditions. To test this hypothesis, we treated *Pex2* ^*−/−*^ BMMCs with niacin for 48 h to reduce FFAs (Figure 3B) and then we stimulated the cells. Treatments with niacin reduced FFAs in *Pex2* ^*−/−*^ BMMCs at rest to the amount observed in WT. Moreover, after IgE stimulation, niacin treatment recapitulated the increase FFAs observed in IgE stimulated WT BMMC (Figure 3B). Intriguingly, the amounts of released IL-6 and IL-13 after IgE-mediated activation was rescued to WT levels in *Pex2* ^*−/−*^ BMMCs upon 48 h treatment with niacin (Figures 3C,D). This result provides a link between the cellular FFAs milieu, regulated by peroxisomes, and cytokine release by MCs.

We affected cellular FFAs by treatment with thioridazine, a small molecule that causes accumulation of FFAs (Van den Branden and Roels, 1985; Shi et al., 2012). Similar to *Pex2* ^*−/−*^ BMMCs, thioridazine-treated WT BMMCs present high amount of cellular FFAs. Upon IgE stimulation, thioridazine-treated WT BMMCs secreted lower amount of IL-6 (Figure 3E) and IL-13 (Figure 3F) compared to untreated WT BMMCs. Of note, all the treatments used to stimulate MCs and/or to manipulate cellular FFAs did not affect cell viability (Supplementary Figure S2D).

**FIGURE 4:**
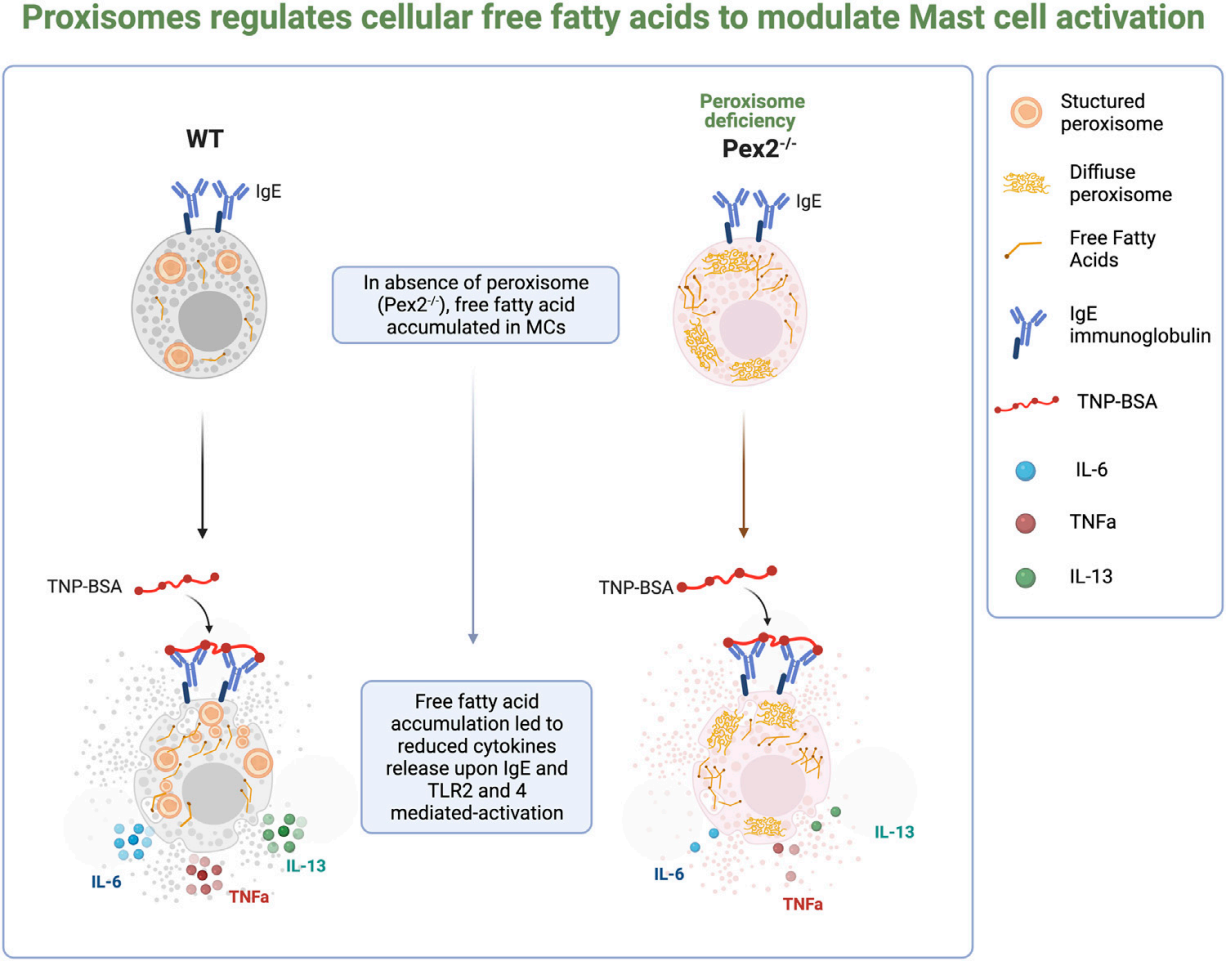
Schematic summary of the finding from the study.

These results indicated that peroxisomal control of cellular FFAs is required in MCs to respond to and participate in inflammatory signaling cascades (Figure 4).

## Discussion

Peroxisomes are ubiquitous organelles with a central role in lipid metabolism and ROS production and scavenging (Schrader and Fahimi, 2006). Peroxisomes have been recognized as organelles of immunity with central immunometabolic and signaling functions to regulate immune response to pathogens (Sychev et al., 2017; Xu et al., 2017; Cook et al., 2019; Merkling et al., 2019). We have previously shown the central role of peroxisome in macrophage-mediated host defense (Di Cara et al., 2017; Nath et al., 2022). The present study aimed to define the role of peroxisome metabolism in the context of TLR and IgE-mediated activation of MCs. In the immune compartment, peroxisome-derived lipids have been shown to be involved in the development, survival and functions of multiple innate and adaptive immune effector cells (Brutkiewicz and Dent, 2012; Facciotti et al., 2012; Di Cara, 2020).

Lipids are important mediators of mast cell immune functions (Nakamura et al., 1991; Austen, 2005; Dichlberger et al., 2013). However, the role of peroxisomes in regulating MCs development and activation is unknown. Here we determined that lack of functional peroxisomes in MCs did not alter their maturation, morphology, or granulation. We report that an increase in peroxisome number occurs in MCs upon IgE-mediated activation, indicating the involvement of peroxisomes or peroxisome metabolism in mast cell responses. Peroxisomes have been shown to mobilize and to metabolically support activation during viral infection (Cook et al., 2019) as well as phagocytosis by macrophages (Eguchi et al., 1979; Di Cara et al., 2017). While the number of peroxisomes are increased during IgE-mediated activation, MC degranulation remained unchanged in absence of peroxisomes, suggesting that peroxisomes might support other mast cell-specific responses to IgE stimulation but not the degranulation.

Interestingly, in our study, an absence of functional peroxisomes in MCs led to a significant decrease in TLR2 and TLR4-mediated IL-6, IL-13 and TNF cytokine production compared to WT MCs. In potential contrast, Vijayan et al. showed that peroxisome induction with 4-phenyl butyric acid in macrophages dampened their IL-6, IL-12 and TNF production in response to TLR4-mediated activation, suggesting an anti-inflammatory role for peroxisomes in these cells (Vijayan et al., 2017). On the other hand, Nath et al., reported a deficiency in IL-6, IL-1β, and TNF secretion in response to TLR1/2 and TLR4-mediated activation in *Pex2* ^*−/−*^ macrophages. When stimulated with IgE/antigen MCs exhibited a decrease in IL-6, IL-13, and TNF release supporting the hypothesis that peroxisomes might have pro or anti-inflammatory functions in different myeloid cells. Of note, peroxisome dysfunction also impacted cytokine release following calcium ionophore stimulation but to a lesser extent. All together our results indicated a stimuli-dependent role of peroxisomes in MC activation.

Peroxisomes main metabolic functions include β-oxidation of very long chain fatty acids and metabolism of ROS. We previously showed that lack of functional peroxisomes affects cellular H_2_O_2_-mediated signaling that controls uptake of pathogens by phagocytosis and activation of NF-κB (Di Cara et al., 2017). While ROS catabolism was unaffected in MCs which lacked functional peroxisomes, as H_2_O_2_ amounts were unchanged, cellular amounts of FFAs were altered in the absence of peroxisomes under both unstimulated and stimulated conditions. Upon activation, changes in lipid composition are expected in immune cells (Sadik and Luster, 2012; Lodhi et al., 2015) as lipids are crucial mediators for cell signaling as well as regulation of inflammation (Sadik and Luster, 2012). Wild-type MCs exhibited an increased FFAs level after IgE-mediated activation while this increase was not observed in the absence of peroxisomes, highlighting the importance of peroxisomes in lipid dynamics in MCs. These observations indicate an excess of lipids in the absence of functional peroxisomes in MCs. Remarkably, when peroxisome deficient MCs were treated, prior to activation, with niacin, a FFAs scavenger (Papaliodis et al., 2008), their cytokine release function was restored, indicating a restored FFAs turnover with the treatment. Furthermore, we demonstrated that peroxisome metabolism has a role in regulating FFAs cellular amounts observed during IgE activation of MCs. This FFAs regulation appeared a key mechanism of peroxisome activity in MCs. In fact, treatment of mast cells with thioridazine, a small molecule that triggers accumulation of cellular FFAs, recapitulated the phenotype observed in *Pex2* ^*−/−*^ cells. The roles of peroxisomal FFAs homeostasis have not been extensively studied in immune cells, nevertheless, a few studies showed that thioridazine treatment decreased TLR mediated activation in macrophages (Baig et al., 2018; Ganguli et al., 2019) and in T cells reducing *in vitro* murine Treg cell polarization while no effects were found on Th1 or Th17 cells (Moreno-Fernandez et al., 2018).

MCs are critical, tissue-resident sentinel cells with a wide range of impacts on innate immunity and the mobilisation of effective acquired immune responses to infection, as well as impacts on cancer development and anti-cancer immunity. They are rich at sites that interface with the external environment such as skin and mucosae and also elevated around many types of solid tumours. They have been implicated in effective local mobilisation of immune responses to a number of parasitic, bacterial, viral and fungal challenges, in some cases, these include the generation of ROS, as well as degranulation or selective cytokine and chemokine production. These studies suggest that many aspects of such sentinel functions against infection, such as the production of pro-inflammatory cytokines might be modulated by peroxisomal activity. Mast cell responses are known to be modulated by lipid mediators, endocannabinoids and FFAs (Abdel-Majid and Marshall, 2004; Hagemann et al., 2019). The current study showed the importance of peroxisome-mediated lipid metabolism in MCs and indicates that proper regulation of FFAs modulates MCs activation and cytokine production. Our work and recent studies (Eguchi et al., 1979; Singh et al., 2004; Di Cara et al., 2017, 2019; Vijayan et al., 2017; Nath et al., 2022) revealing peroxisome involvement in immune processes, provide a new avenue for therapeutic targeting. Such interventions, focused on MCs, may allow local modulation of immune and inflammatory events in specific mast cell-rich tissues such as the skin, airways or tumour microenvironment. The selective nature of the impact of peroxisomes on mast cell function may suggest new pharmacological approaches to modify cytokine production, such as that observed in chronic inflammatory sites without limiting the acute degranulation events necessary for rapid recruitment of immune effector cells and dendritic cell mobilisation at the very earliest stages of infection. The role of peroxisomes in MCs in regulating allergic disease remains unclear, however the impact of peroxisome defects on IL-13 production may also suggest that such organelle function could be targeted in the context of chronic allergic inflammation.

## Data Availability

The original contributions presented in the study are included in the article/Supplementary Material, further inquiries can be directed to the corresponding authors.

## References

[B1] Abdel-Majid R. M. Marshall J. S. (2004). Prostaglandin E2Induces Degranulation-independent Production of Vascular Endothelial Growth Factor by Human Mast Cells. J. Immunol. 172, 1227–1236. 10.4049/jimmunol.172.2.1227 14707101

[B2] Abraham S. N. St. John A. L. (2010). Mast Cell-Orchestrated Immunity to Pathogens. Nat. Rev. Immunol. 10, 440–452. 10.1038/nri2782 20498670 PMC4469150

[B3] Agier J. Pastwińska J. Brzezińska-Błaszczyk E. (2018). An Overview of Mast Cell Pattern Recognition Receptors. Inflamm. Res. 67, 737–746. 10.1007/s00011-018-1164-5 29909493 PMC6096630

[B4] Aldan J. T. Jansen C. Speck M. Maaetoft-Udsen K. Cordasco E. A. Faiai M. U. (2019). Insulin-induced Lipid Body Accumulation Is Accompanied by Lipid Remodelling in Model Mast Cells. Adipocyte 8, 265–279. 10.1080/21623945.2019.1636624 31311389 PMC6768188

[B5] Baig M. S. Saqib U. Rajpoot S. Srivastava M. Naim A. Liu D. (2018). Repurposing Thioridazine (TDZ) as an Anti-inflammatory Agent. Sci. Rep. 8, 12471. 10.1038/s41598-018-30763-5 30127400 PMC6102213

[B6] Beach A. Burstein M. T. Richard V. R. Leonov A. Levy S. Titorenko V. I. (2012). Integration of Peroxisomes into an Endomembrane System that Governs Cellular Aging. Front. Physio. 3, 283. 10.3389/fphys.2012.00283 PMC3424522 22936916

[B7] Bedard K. Krause K.-H. (2007). The NOX Family of ROS-Generating NADPH Oxidases: Physiology and Pathophysiology. Physiol. Rev. 87, 245–313. 10.1152/physrev.00044.2005 17237347

[B8] Bettencourt I. A. Powell J. D. (2017). Targeting Metabolism as a Novel Therapeutic Approach to Autoimmunity, Inflammation, and Transplantation. J. Immunol. 198, 999–1005. 10.4049/jimmunol.1601318 28115589 PMC5300074

[B9] Blander J. M. Sander L. E. (2012). Beyond Pattern Recognition: Five Immune Checkpoints for Scaling the Microbial Threat. Nat. Rev. Immunol. 12, 215–225. 10.1038/nri3167 22362354

[B74] Boncompain G. Muller C. Meas-Yedid V. Schmitt-Kopplin P. Lazarow P. B. Subtil A. (2014). The Intracellular Bacteria Chlamydia Hijack Peroxisomes and Utilize Their Enzymatic Capacity to Produce Bacteria-Specific Phospholipids. PLoS One 9, e86196 24465954 10.1371/journal.pone.0086196 PMC3900481

[B10] Boyce J. A. (2005). Eicosanoid Mediators of Mast Cells: Receptors, Regulation of Synthesis, and Pathobiologic Implications. Chem. Immunol. Allergy 87, 59–79. 10.1159/000087571 16107763

[B11] Braverman N. E. D'Agostino M. D. Maclean G. E. (2013). Peroxisome Biogenesis Disorders: Biological, Clinical and Pathophysiological Perspectives. Dev. Disabil. Res. Rev. 17, 187–196. 10.1002/ddrr.1113 23798008

[B12] Brutkiewicz R. R. Dent A. L. (2012). Lipids-Я-Us: Peroxisome Generation of iNKT Ligands. Nat. Immunol. 13, 435–436. 10.1038/ni.2288 22513327

[B13] Colasante C. Chen J. Ahlemeyer B. Baumgart-Vogt E. (2015). Peroxisomes in Cardiomyocytes and the Peroxisome/Peroxisome Proliferator-Activated Receptor-Loop. Thromb. Haemost. 113, 452–463. 10.1160/TH14-06-0497 25608554

[B14] Cook K. C. Moreno J. A. Jean Beltran P. M. Cristea I. M. (2019). Peroxisome Plasticity at the Virus-Host Interface. Trends Microbiol. 27, 906–914. 10.1016/j.tim.2019.06.006 31331665 PMC6857447

[B15] Di Cara F. Andreoletti P. Trompier D. Vejux A. Bülow M. H. Sellin J. (2019). Peroxisomes in Immune Response and Inflammation. Int. J. Mol. Sci. 20, 3877. 10.3390/ijms20163877 PMC6721249 31398943

[B16] Di Cara F. (2020). Peroxisomes in Host Defense. Plos Pathog. 16, e1008636. 10.1371/journal.ppat.1008636 32614930 PMC7331978

[B17] Di Cara F. Sheshachalam A. Braverman N. E. Rachubinski R. A. Simmonds A. J. (2017). Peroxisome-Mediated Metabolism Is Required for Immune Response to Microbial Infection. Immunity 47, 93–106. 10.1016/j.immuni.2017.06.016 28723556

[B18] Dichlberger A. Kovanen P. T. Schneider W. J. (2013). Mast Cells: from Lipid Droplets to Lipid Mediators. Clin. Sci. (Lond) 125, 121–130. 10.1042/CS20120602 23577635 PMC3631086

[B19] Dixit E. Boulant S. Zhang Y. Lee A. S. Y. Odendall C. Shum B. (2010). Peroxisomes Are Signaling Platforms for Antiviral Innate Immunity. Cell 141, 668–681. 10.1016/j.cell.2010.04.018 20451243 PMC3670185

[B20] Dowds C. M. Kornell S.-C. Blumberg R. S. Zeissig S. (2014). Lipid Antigens in Immunity. Biol. Chem. 395, 61–81. 10.1515/hsz-2013-0220 23999493 PMC4128234

[B21] Eguchi M. Sannes P. L. Spicer S. S. (1979). Peroxisomes of Rat Peritoneal Macrophages during Phagocytosis. Am. J. Pathol. 95, 281 453318 PMC2042341

[B22] Facciotti F. Ramanjaneyulu G. S. Lepore M. Sansano S. Cavallari M. Kistowska M. (2012). Peroxisome-derived Lipids Are Self Antigens that Stimulate Invariant Natural Killer T Cells in the Thymus. Nat. Immunol. 13, 474–480. 10.1038/ni.2245 22426352

[B23] Faust P. L. (2003). Abnormal Cerebellar Histogenesis inPEX2 Zellweger Mice Reflects Multiple Neuronal Defects Induced by Peroxisome Deficiency. J. Comp. Neurol. 461, 394–413. 10.1002/cne.10699 12746876

[B24] Faust P. L. Hatten M. E. (1997). Targeted Deletion of the PEX2 Peroxisome Assembly Gene in Mice Provides a Model for Zellweger Syndrome, a Human Neuronal Migration Disorder. J. Cell Biol 139, 1293–1305. 10.1083/jcb.139.5.1293 9382874 PMC2140200

[B25] Frank Austen K. (2005). The Mast Cell and the Cysteinyl Leukotrienes. Novartis Found Symp. 271, 166–178. 10.1002/9780470033449.ch13 16605134

[B26] Fransen M. Nordgren M. Wang B. Apanasets O. (2012). Role of Peroxisomes in ROS/RNS-metabolism: Implications for Human Disease. Biochim. Biophys. Acta (Bba) - Mol. Basis Dis. 1822, 1363–1373. 10.1016/j.bbadis.2011.12.001 22178243

[B27] Fransen M. Nordgren M. Wang B. Apanasets O. Van Veldhoven P. P. (2013). Aging, Age-Related Diseases and Peroxisomes. Subcell Biochem. 69, 45–65. 10.1007/978-94-007-6889-5_3 23821142

[B28] Ganguli G. Mukherjee U. Sonawane A. (2019). Peroxisomes and Oxidative Stress: Their Implications in the Modulation of Cellular Immunity during Mycobacterial Infection. Front. Microbiol. 10, 1121. 10.3389/fmicb.2019.01121 31258517 PMC6587667

[B29] Germain V. Rylott E. L. Larson T. R. Sherson S. M. Bechtold N. Carde J.-P. (2001). Requirement for 3-Ketoacyl-CoA Thiolase-2 in Peroxisome Development, Fatty Acid β-oxidation and Breakdown of Triacylglycerol in Lipid Bodies of Arabidopsis Seedlings. Plant J. 28, 1–12. 10.1046/j.1365-313x.2001.01095.x 11696182

[B30] Greineisen W. E. Maaetoft-Udsen K. Speck M. Balajadia J. Shimoda L. M. N. Sung C. (2015). Chronic Insulin Exposure Induces ER Stress and Lipid Body Accumulation in Mast Cells at the Expense of Their Secretory Degranulation Response. PLoS One 10, e0130198. 10.1371/journal.pone.0130198 26263026 PMC4532411

[B31] Hagemann P. M. Nsiah-Dosu S. Hundt J. E. Hartmann K. Orinska Z. (2019). Modulation of Mast Cell Reactivity by Lipids: The Neglected Side of Allergic Diseases. Front. Immunol. 10, 1174. 10.3389/fimmu.2019.01174 31191542 PMC6549522

[B32] Hanes M. R. Giacomantonio C. A. Marshall J. S. (2021). Mast Cells and Skin and Breast Cancers: A Complicated and Microenvironment-dependent Role. Cells 10, 986. 10.3390/cells10050986 33922465 PMC8146516

[B33] Hubler M. J. Kennedy A. J. (2016). Role of Lipids in the Metabolism and Activation of Immune Cells. J. Nutr. Biochem. 34, 1–7. 10.1016/j.jnutbio.2015.11.002 27424223 PMC5694687

[B34] Jain R. Tikoo S. Weninger W. (2019). Mast Cell Granules: Modulating Adaptive Immune Response Remotely. J. Allergy Clin. Immunol. 143, 1731–1733. 10.1016/j.jaci.2018.11.029 30557603

[B35] Knoblach B. Ishida R. Hobman T. C. Rachubinski R. A. (2021). Peroxisomes Exhibit Compromised Structure and Matrix Protein Content in SARS-CoV-2-Infected Cells. MBoC 32, 1273–1282. 10.1091/mbc.E21-02-0074 34010015 PMC8351553

[B36] Komi D. E. A. Redegeld F. A. (2020). Role of Mast Cells in Shaping the Tumor Microenvironment. Clinic Rev. Allerg Immunol. 58, 313–325. 10.1007/s12016-019-08753-w PMC7244463 31256327

[B37] Leal-Berumen I. Conlon P. Marshall J. S. (1994). IL-6 Production by Rat Peritoneal Mast Cells Is Not Necessarily Preceded by Histamine Release and Can Be Induced by Bacterial Lipopolysaccharide. J. Immunol. 152, 5468 7514639

[B38] Lim H.-K. Choi Y.-A. Park W. Lee T. Ryu S. H. Kim S.-Y. (2003). Phosphatidic Acid Regulates Systemic Inflammatory Responses by Modulating the Akt-Mammalian Target of Rapamycin-P70 S6 Kinase 1 Pathway. J. Biol. Chem. 278, 45117–45127. 10.1074/jbc.M303789200 12960176

[B39] Liu J. Lu W. Shi B. Klein S. Su X. (2019). Peroxisomal Regulation of Redox Homeostasis and Adipocyte Metabolism. Redox Biol. 24, 101167. 10.1016/j.redox.2019.101167 30921635 PMC6434164

[B40] Lodhi I. J. Semenkovich C. F. (2014). Peroxisomes: a Nexus for Lipid Metabolism and Cellular Signaling. Cell Metab. 19, 380–392. 10.1016/j.cmet.2014.01.002 24508507 PMC3951609

[B41] Lodhi I. J. Wei X. Yin L. Feng C. Adak S. Abou-Ezzi G. (2015). Peroxisomal Lipid Synthesis Regulates Inflammation by Sustaining Neutrophil Membrane Phospholipid Composition and Viability. Cell Metab. 21, 51–64. 10.1016/j.cmet.2014.12.002 25565205 PMC4287274

[B42] Marshall J. S. (2004). Mast-cell Responses to Pathogens. Nat. Rev. Immunol. 4, 787–799. 10.1038/nri1460 15459670

[B43] McCurdy J. D. Olynych T. J. Maher L. H. Marshall J. S. (2003). Cutting Edge: Distinct Toll-like Receptor 2 Activators Selectively Induce Different Classes of Mediator Production from Human Mast Cells. J. Immunol. 170, 1625–1629. 10.4049/jimmunol.170.4.1625 12574323

[B44] Mendoza R. P. Anderson C. C. Fudge D. H. Roede J. R. Brown J. M. (2021). Metabolic Consequences of IgE- and Non-IgE-mediated Mast Cell Degranulation. J. Immunol. 207, 2637–2648. 34732470 10.4049/jimmunol.2001278 PMC8612977

[B45] Merkling S. H. Riahi H. Overheul G. J. Schenck A. van Rij R. P. (2019). Peroxisome-associated Sgroppino Links Fat Metabolism with Survival after RNA Virus Infection in Drosophila. Sci. Rep. 9, 2065–2112. 10.1038/s41598-019-38559-x 30765784 PMC6375949

[B46] Miao H. Ou J. Ma Y. Guo F. Yang Z. Wiggins M. (2014). Macrophage CGI-58 Deficiency Activates ROS-Inflammasome Pathway to Promote Insulin Resistance in Mice. Cell Rep. 7, 223–235. 10.1016/j.celrep.2014.02.047 24703845 PMC4040312

[B47] Moon T. C. Befus A. D. Kulka M. (2014). Mast Cell Mediators: Their Differential Release and the Secretory Pathways Involved. Front. Immunol. 5, 569. 10.3389/fimmu.2014.00569 25452755 PMC4231949

[B48] Moreno-Fernandez M. E. Giles D. A. Stankiewicz T. E. Sheridan R. Karns R. Cappelletti M. (2018). Peroxisomal β-oxidation Regulates Whole Body Metabolism, Inflammatory Vigor, and Pathogenesis of Nonalcoholic Fatty Liver Disease. JCI Insight 3, e93626. 10.1172/jci.insight.93626 PMC5926941 29563328

[B49] Nakamura T. Fonteh A. N. Hubbard W. C. Triggiani M. Inagaki N. Ishizaka T. (1991). Arachidonic Acid Metabolism during Antigen and Ionophore Activation of the Mouse Bone Marrow Derived Mast Cell. Biochim. Biophys. Acta. 1085, 191–200. 10.1016/0005-2760(91)90094-x 1892888

[B50] Nath A. S. Parsons B. D. Makdissi S. Chilvers R. L. Mu Y. Weaver C. M. (2022). Modulation of the Cell Membrane Lipid Milieu by Peroxisomal β-oxidation Induces Rho1 Signaling to Trigger Inflammatory Responses. Cell Rep. 38, 110433. 10.1016/j.celrep.2022.110433 35235794

[B51] O'Neill L. A. J. Kishton R. J. Rathmell J. (2016). A Guide to Immunometabolism for Immunologists. Nat. Rev. Immunol. 16, 553–565. 10.1038/nri.2016.70 27396447 PMC5001910

[B52] Oldford S. A. Haidl I. D. Howatt M. A. Leiva C. A. Johnston B. Marshall J. S. (2010). A Critical Role for Mast Cells and Mast Cell-Derived IL-6 in TLR2-Mediated Inhibition of Tumor Growth. J. Immunol. 185, 7067–7076. 10.4049/jimmunol.1001137 21041732

[B53] Papaliodis D. Boucher W. Kempuraj D. Michaelian M. Wolfberg A. House M. (2008). Niacin-induced "Flush" Involves Release of Prostaglandin D2from Mast Cells and Serotonin from Platelets: Evidence from Human Cells *In Vitro* and an Animal Model. J. Pharmacol. Exp. Ther. 327, 665–672. 10.1124/jpet.108.141333 18784348

[B54] Puertollano M. A. Pablo M. A. Ã lvarez de Cienfuegos G. (2001). Immunomodulatory Effects of Dietary Lipids Alter Host Natural Resistance of Mice toListeria Monocytogenesinfection. FEMS Immunol. Med. Microbiol. 32, 47–52. 10.1111/j.1574-695X.2001.tb00533.x 11750222

[B55] Sadik C. D. Luster A. D. (2012). Lipid-cytokine-chemokine Cascades Orchestrate Leukocyte Recruitment in Inflammation. J. Leukoc. Biol. 91, 207–215. 10.1189/jlb.0811402 22058421 PMC3290425

[B56] Schrader M. Fahimi H. D. (2006). Peroxisomes and Oxidative Stress. Biochim. Biophys. Acta. 1763, 1755–1766. 10.1016/j.bbamcr.2006.09.006 17034877

[B57] Schrader M. Kamoshita M. Islinger M. (2020). Organelle Interplay-Peroxisome Interactions in Health and Disease. J. Inherit. Metab. Dis. 43, 71–89. 10.1002/jimd.12083 30864148 PMC7041636

[B58] Schwartz L. B. Austen K. F. Wasserman S. I. (1979). Immunologic Release of Beta-Hexosaminidase and Beta-Glucuronidase from Purified Rat Serosal Mast Cells. J. Immunol. 123, 1445 479592

[B59] Shi R. Zhang Y. Shi Y. Shi S. Jiang L. (2012). Inhibition of Peroxisomal β-oxidation by Thioridazine Increases the Amount of VLCFAs and Aβ Generation in the Rat Brain. Neurosci. Lett. 528, 6–10. 10.1016/j.neulet.2012.08.086 22985512

[B60] Singh I. Paintlia A. S. Khan M. Stanislaus R. Paintlia M. K. Haq E. (2004). Impaired Peroxisomal Function in the central Nervous System with Inflammatory Disease of Experimental Autoimmune Encephalomyelitis Animals and protection by Lovastatin Treatment. Brain Res. 1022, 1–11. 10.1016/j.brainres.2004.06.059 15353207

[B61] Smith J. J. Aitchison J. D. (2013). Peroxisomes Take Shape. Nat. Rev. Mol. Cell Biol 14, 803–817. 10.1038/nrm3700 24263361 PMC4060825

[B62] Sychev Z. E. Hu A. DiMaio T. A. Gitter A. Camp N. D. Noble W. S. (2017). Integrated Systems Biology Analysis of KSHV Latent Infection Reveals Viral Induction and reliance on Peroxisome Mediated Lipid Metabolism. Plos Pathog. 13, e1006256. 10.1371/journal.ppat.1006256 28257516 PMC5352148

[B63] Szilard R. K. Titorenko V. I. Veenhuis M. Rachubinski R. A. (1995). Pay32p of the Yeast Yarrowia Lipolytica Is an Intraperoxisomal Component of the Matrix Protein Translocation Machinery. J. Cell Biol 131, 1453–1469. 10.1083/jcb.131.6.1453 8522603 PMC2120665

[B64] Tertian G. Yung Y. P. Guy-Grand D. Moore M. A. (1981). Long-term *In Vitro* Culture of Murine Mast Cells. I Description of a Growth Factor-dependent Culture Technique. J. Immunol. 127, 788 7019332

[B65] Theoharides T. C. Tsilioni I. Ren H. (2019). Recent Advances in Our Understanding of Mast Cell Activation - or Should it Be Mast Cell Mediator Disorders? Expert Rev. Clin. Immunol. 15, 639–656. 10.1080/1744666X.2019.1596800 30884251 PMC7003574

[B66] Trompier D. Vejux A. Zarrouk A. Gondcaille C. Geillon F. Nury T. Savary S. Lizard G. (2014). Brain peroxisomes. Biochimie 98, 102–110. 10.1016/j.biochi.2013.09.009 24060512

[B67] Tunaru S. Kero J. Schaub A. Wufka C. Blaukat A. Pfeffer K. (2003). PUMA-G and HM_74_ Are Receptors for Nicotinic Acid and Mediate its Anti-lipolytic Effect. Nat. Med. 9, 352–355. 10.1038/nm824 12563315

[B68] Van den Branden C. Roels F. (1985). Thioridazine: a Selective Inhibitor of Peroxisomal β-oxidation *In Vivo* . FEBS Lett. 187, 331–333. 10.1016/0014-5793(85)81270-9 4018269

[B69] Vijayan V. Srinu T. Karnati S. Garikapati V. Linke M. Kamalyan L. (2017). A New Immunomodulatory Role for Peroxisomes in Macrophages Activated by the TLR4 Ligand Lipopolysaccharide. J. Immunol. 198, 2414–2425. 10.4049/jimmunol.1601596 28179495

[B70] Wanders R. J. A. Waterham H. R. (2006). Biochemistry of Mammalian Peroxisomes Revisited. Annu. Rev. Biochem. 75, 295–332. 10.1146/annurev.biochem.74.082803.133329 16756494

[B71] Wang X. Kulka M. (2015). n-3 Polyunsaturated Fatty Acids and Mast Cell Activation. J. Leukoc. Biol. 97, 859–871. 10.1189/jlb.2RU0814-388R 25765678

[B72] Wernersson S. Pejler G. (2014). Mast Cell Secretory Granules: Armed for Battle. Nat. Rev. Immunol. 14, 478–494. 10.1038/nri3690 24903914

[B73] Xu Z. Asahchop E. L. Branton W. G. Gelman B. B. Power C. Hobman T. C. (2017). MicroRNAs Upregulated during HIV Infection Target Peroxisome Biogenesis Factors: Implications for Virus Biology, Disease Mechanisms and Neuropathology. Plos Pathog. 13, e1006360. 10.1371/journal.ppat.1006360 28594894 PMC5464672

