# Supplementary figures and images for: Peroxisomes Regulate Cellular Free Fatty Acids to Modulate Mast Cell TLR2, TLR4, and IgE-Mediated Activation
Source: Front Cell Dev Biol. 2022 May 13;10:856243. doi: 10.3389/fcell.2022.856243 (PMC9215104; doi:10.3389/fcell.2022.856243)

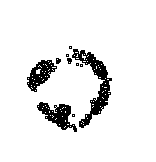

Supplement: Supplementary file 1 [file Image6.TIF]

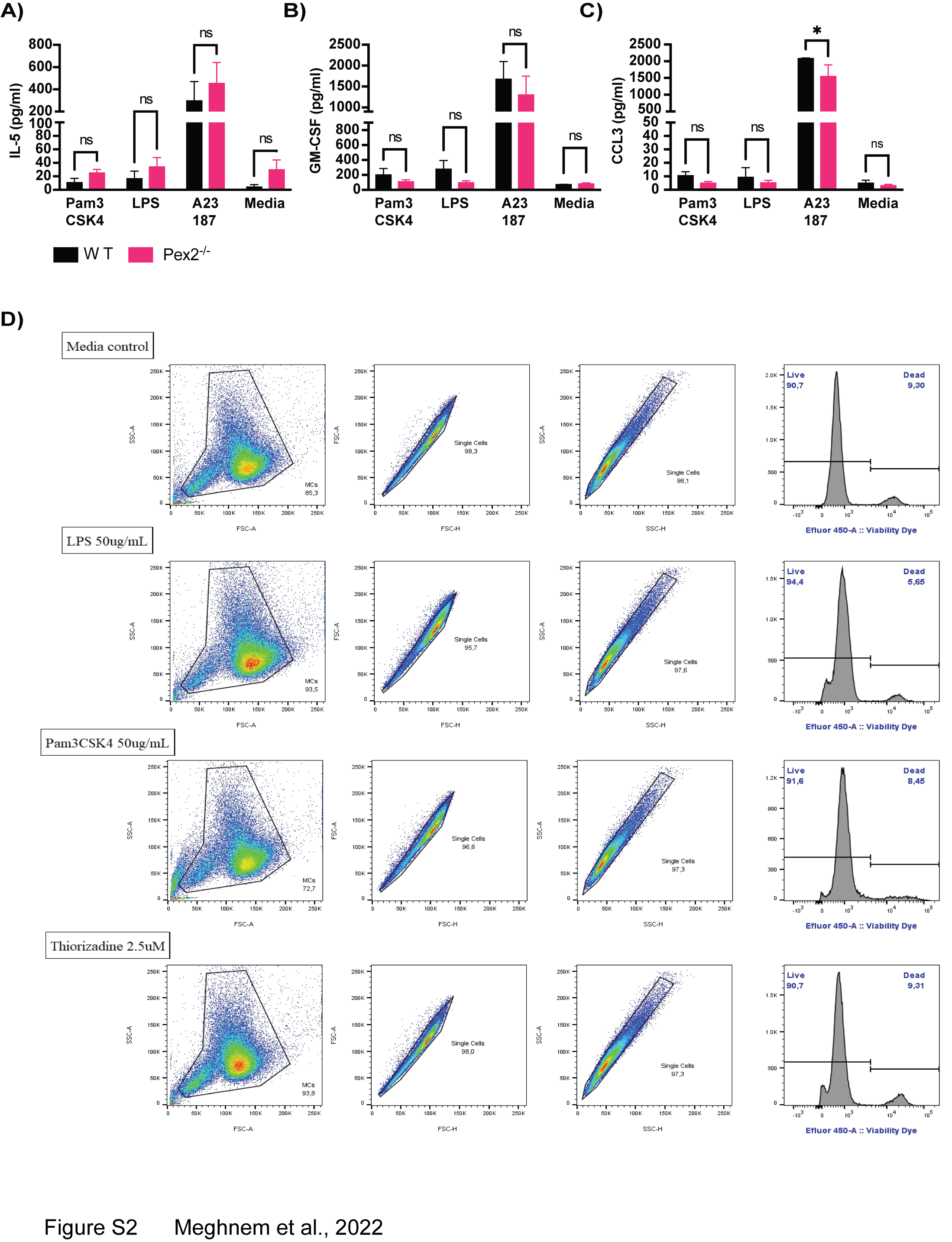

Supplement: Supplementary file 4 [file Image2.TIF]

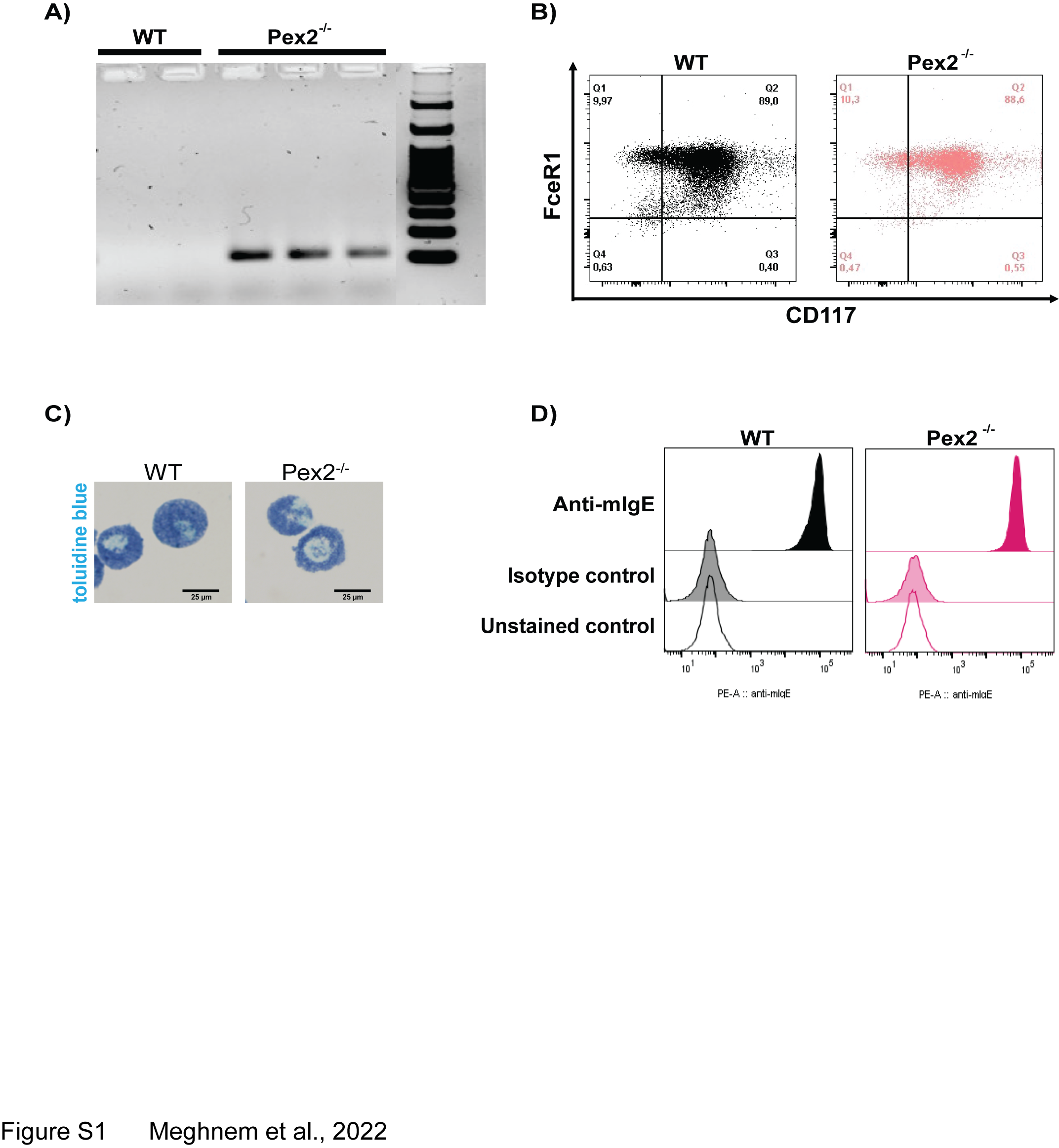

Supplement: Supplementary file 5 [file Image1.TIF]
